# From New York

**Published:** 1892-12

**Authors:** Wm. L. Russell

**Affiliations:** 151 East 50th St.; New York


### *CORRESPONDENCE.*

NEW YORK, November 12, 1892.

### INTUBATION VERSUS TRACHEOTOMY.

In a recent number of the *Medical News*, Dr. Lovett, of the Boston City Hospital, published an article in which he gave the statistics of these two operations as they appeared in the records of the hospital. The result was rather against intubation and in favor of tracheotomy. Considerable interest was aroused here, and the subject came up for discussion at the October meeting of the Academy.

In opening the discussion Dr. O'Dwyer said that these operations were performed so seldom for other conditions, that the term acute laryngeal stenosis, could be regarded as meaning croup. Ninety per cent. of these cases were fatal if not interfered with. If stenosis pure and simple was the only condition present, there would be no failures from intubation in skillful hands. Bronchial plugging, broncho-pneumonia, nephritis and paralysis were some of the additional factors operating to produce death.

The high mortality at the Boston City Hospital could be explained by the fact that the operation was done by the members of the house staff in turn. There should be no failures in accomplishing the intubation. In cases in which it was reported that the tube had been found in the bronchus, force enough must have been used to fracture the cricoid cartilage. No one should attempt to intubate in the living subject until practice on the cadaver had demonstrated his ability to insert or withdraw the tube in five seconds. Lacerations caused by the introduction of the tube were liable to produce serious complications. The difficulty in feeding experienced by some was overcome by placing the child in the Casselbury position, the head being held well back, the child lying on the back. Tracheotomy required no great technical skill in its performance, but careful nursing was all important. The recovery rate from tracheotomy at the Boston City Hospital was twenty-nine per cent., while that after intubation was twenty per cent. At the Willard Parker Hospital, however, in this city, the recovery rate in 186 cases intubated during the past three years was thirty-eight per cent. On the whole, he believed that intubation accomplished all that tracheotomy did without the unpleasant features of the latter.

Dr. L. S. Pilcher agreed with Dr. O'Dwyer in regarding all cases of acute stenosis of the larynx as due to croup, and in comparing tracheotomy and intubation he thought it necessary to take into consideration the effects of the general disease which was operating in conjunction with the stenosis. Both the operations would relieve the stenosis equally well. In the matter o blood aeration all that was necessary was accomplished by intubation, the air being also rendered warm and moist in its passage through the nose. The narrowness of the lumen of the tube, however, while increasing the force of the cough was not favorable to the expulsion of the membrane. The crises liable to result from this circumstance were avoided in tacheotomy. The matter of local cleanliness was as important in the larynx as in pharynx, but could not be accomplished as well after intubation as after tracheotomy which facilitated the escape of membrane and secretions, and allowed of the employment of cleansing measures. He believed that the embarrassment of deglutition after intubation was very serious, the publications on the subject being optimistic. No such disadvantage occurred after tracheotomy. The deductions from statistics were liable to be misleading, as intubation was frequently done in less urgent cases than any treated by tracheotomy. The conditions for combating the general infection were provided best by tracheotomy. The difficulties belonging to the operation appertained largely to the after treatment. To meet this difficulty, isolated wards should be provided in the hospital to which croup cases could be sent, and where they would receive competent after treatment. Under such circumstances he believed that tracheotomy would prove to be the most efficient operation.

Dr. G. W. Gay, of Boston, said that neither operation had much effect on the extension of the disease, this being determined by the degree of infection. The stenosis was the only point to consider. Intubation gave immediate and satisfactory relief in nine cases out of ten, and many of the failures were due to faulty introduction. The operation was not perfect, but it had found a permanent place. Dysphagia was the most serious objection, the short time required for its performance, and the absence of shock being strong points in its favor. It was the preferable operation in all cases under seven years of age. The consent of the parents was easily obtained, the after treatment was not so important as after tracheotomy, no anæsthetic was necessary, and no wound was made.

### APPENDICITIS.

We have not yet heard the last of this subject, and there are grounds for believing that knowledge concerning it has accumulated nearly to the point at which definite rules for the management of cases can be formulated. There will always be differences of opinion, of course, regarding the exact time to operate in individual cases, but the advisability or inadvisability of an early operation, and the indications for an operation are gradually becoming settled.

Dr. J. A. Wyeth read a paper on the subject at the Surgical Section last evening. He advocated early operation and the removal of the appendix in every instance. Dr. B. F. Curtis did not agree with Dr. Wyeth's views regarding the removal of the appendix. When there were adhesions and danger of opening up the general peritoneal cavity, the great danger of general infection could not be ignored. Dr. Janeway said that in many cases it was difficult to decide regarding the advisability of operation. If the disease existed in a good subject, not too old or too fat, and with good heart and vessels, an early operation was always advisable. There were sometimes difficulties in diagnosis. He had seen instances in which pneumonia, empyema and intercostal neuralgia had all been called appendicitis, the pain and even tenderness, being referred to the region of the appendix. On the other hand the pain of appendicitis may be referred to the right hypochondriac region, or may shoot into the testicle and penis simulating renal colic. Fever is not a reliable sign, as the hepatic and renal colic may give rise to a chill and fever. He had known of one case of lumbo-abdominal neuralgia which had been regarded as appendicitis, and an operation done. The appendix was healthy and the operation was painless, save that a hernia which was present was radically operated on and cured. It must not be forgotten too, that there were other causes of peritonitis than appendicitis ; an ovarian abscess was found in one case on operation. It was often difficult to decide whether the peritonitis were general or local. This was especially true of cases which had already been treated by opium which paralyzed the bowels and promoted the retention of gas, causing great tympanites. In cases in which perforation occurred early, when the appendix was free in the abdominal cavity, and signs of general peritonitis advanced rapidly, he believed the prognosis to be bad, whether an operation was done or not.

Dr. Van Valzah thought that cæcitis sometimes occurred independently of appendicitis, and the differential diagnosis of the two was a point needing development. He had seen a number of cases in which the attacks were recurrent get well under saline laxation, rest and other medical measures. He thought these were cases of cæcitis. Dr. Fowler, of Brooklyn, dissented from this view, as he believed appendicitis to be the only condition found. When cases were seen early, and a definite history of colicky pains, nausea and perhaps vomiting, with tenderness over McBurney's point was given, the diagnosis was usually easy. The point of tenderness, however, might not be in the usual situation. In a hundred and fifty cases on which he had operated, there were three in which the seat of tenderness was on the left side, the appendix in these cases being on the left of the median line. In five cases appendicitis was not found, but one case died from the operative interference. He believed that radical measures for the removal of the appendix should be undertaken only when there was no danger of infection, as when the abscess was small.

Dr. Willy Meyer thought that if it could be made a rule to operate at once in all cases many more of those affected during a year would be alive at the end of it than under present conditions. Diagnosis was not always easy ; in one case which he had observed all the symptoms indicated appendicitis, and yet it proved to be a case of renal calculus.

### TELEPHONIC BULLET PROBE.

This ingenious instrument was exhibited to the Section on Surgery by Dr. Girdner. He stated that the ordinary probes used in cases of gunshot injuries detected simply whether a hard or soft body had been touched. The porcelain tips had not worked satisfactorily, as the marks were liable to be rubbed off in the passage of the probe through the tissues. Besides, when the bullet had pursued a tortuous course, it was sometimes impossible to find it. The telephonic probe was intended to overcome all these difficulties. It consisted of an ordinary receiver, such as was used with any telephone, to which was attached two wires three feet in length. To one of the wires was connected an aluminum bulb, and to the other an aluminum probe. In using it, the bulb was placed in the patient's buccal cavity between the cheek and the teeth, the operator then held the receiver to his ear with one hand, while with the other he manipulated the probe in the wound. No contact with the bone or other hard substance than metal would cause any sound to be heard, but as soon as the bullet was touched a grating or scratching sound could be plainly detected. When the wound was tortuous, a steel bulb and a steel probe with a sharp point could be substituted for the aluminum, and the tissues explored until the bullet was detected. No battery was needed, the body of the patient acting in that capacity. It was necessary to observe the precaution of having the bulb and probe of the same metal, and both different from the metal of which the projectile being searched for was composed.

Dr. Girdner's demonstration of the instrument was entirely satisfactory. WM. L. RUSSELL.

151 *East* 50*th St.*

---

### ACNE ROSACEA.

For a case of acne rosacea, Dr. Henry W. Stelwagon gave the following treatment: A saline purgative (sulphate of magnesium or Rochelle salts) to keep the bowels open every day, and the local application of the following lotion:

℞. Zinci sulphat., grains xv.
Potassii sulphuratæ, grains xv.
Aquæ, fluid ounces iv.

M. Sig.—Apply at night.

—*Medical World.*

